# The Final Diagnosis: Colorectal Carcinoma, In the Context of Attenuated Familial Adenomatous Polyposis, Mimicking Pulmonary Carcinoma

**DOI:** 10.7759/cureus.2867

**Published:** 2018-06-23

**Authors:** George S Stoyanov, Lyuben Stoev, Hristo Popov, Ina Kobakova, Deyan L Dzhenkov

**Affiliations:** 1 General and Clinical Pathology/Forensic Medicine and Deontology, Medical University, Varna, BGR; 2 General and Clinical Pathology, Forensic Medicine and Deontology, Medical University, Varna, BGR

**Keywords:** attenuated famillial adenomatous polyposis, colorectal cancer, metastatic disease, autopsy

## Abstract

Pulmonary carcinoma is one of the most common malignant conditions worldwide. The current case presents a patient with lung lesions clinically and radiologically diagnosed as lung cancer, which was not biopsied due to patient’s refusal. The patient was a heavy smoker and prior to the lung lesions, he was diagnosed with chronic obstructive pulmonary disease. Following recurrent hospitalizations, the patient died and he was referred for an autopsy. The autopsy established six lesions in the lung, one in the liver, one in the cerebrum and multiple polyps of the colon, two of which were with a visible invasive growth. The histological sections revealed that the lung, liver, and cerebral lesions were composed of the atypical gland with excessive mucus production. The colorectal specimens revealed benign polyps and colorectal adenocarcinoma. The diagnosis of advanced colorectal adenocarcinoma with multiple metastases in the context of attenuated familial adenomatous polyposis (AFAP) was established due to the combined histological findings, the age of the patient, and the number of benign polyps in the colon.

## Introduction

Pulmonary carcinoma is one of the most common malignant conditions worldwide [1]. There are a myriad of risk factors such as gender, age, race, and exposure to environmental pollutants and tobacco [2]. However, regardless of the patient presentation and exposure to risk factors, histological verification and differentiation are important because of the variety of malignant conditions arising in the lung and it often being a metastatic location for other malignancies [3].

## Case presentation

A 73-year-old male was referred for an autopsy at the Department of Clinical Pathology, St. Marina University Hospital, Varna, Bulgaria from the Intensive Respiratory Unit.

The underlying reason for hospital admission was an exacerbation of a habitually reported dyspnea. The patient was cachexic and he was in a rapidly deteriorating condition. Upon radiological investigation, six formations were found to be present in the lung. The patient reported a severe smoking habit with a history of 50 pack-years.

The patient had multiple hospitalizations in other hospitals in the past; however, he denied biopsy and, therefore, underwent no treatment.

Four days after the hospital admission, the patient died and he was referred for an autopsy to establish the tanatogenetic cause and mechanisms. The clinical diagnosis was bronchogenic cancer rT4NxM1a.

**Figure 1:**
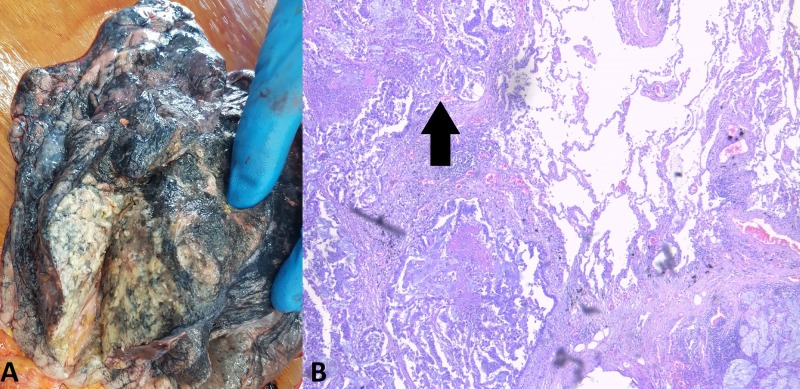
The largest lung lesion. (A) Autopsy finding; (B) histology: hematoxylin and eosin stain, original magnification 100x.

**Figure 2:**
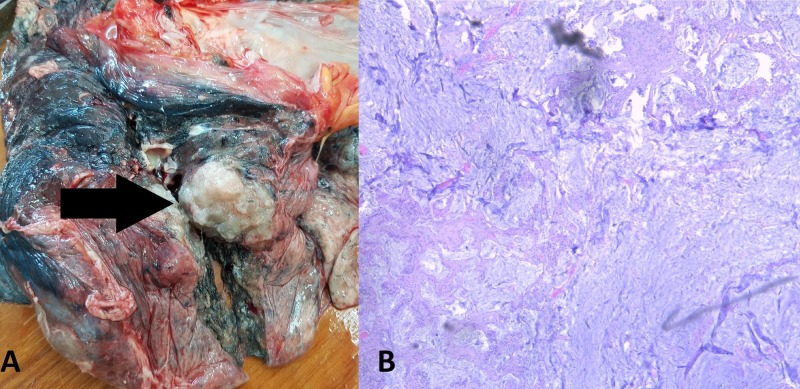
The lung lesion with distinctive appearance when compared to the other lung masses. (A) Autopsy finding; (B) histology: hematoxylin and eosin stain, original magnification 100x, atypical glands with invasive growth into the lung parenchyma (arrow).

Thoracic dissection revealed a total of six well-rounded tumor formations in the lungs, five of which were solid and one was soft and slimy in appearance (Figure 1A-2A). The largest tumor formation measured 8 cm in diameter and the smallest one measured 2 cm in diameter. There was pleural effusion on each thoracic side measuring 200 ml of serous fluid.

**Figure 3:**
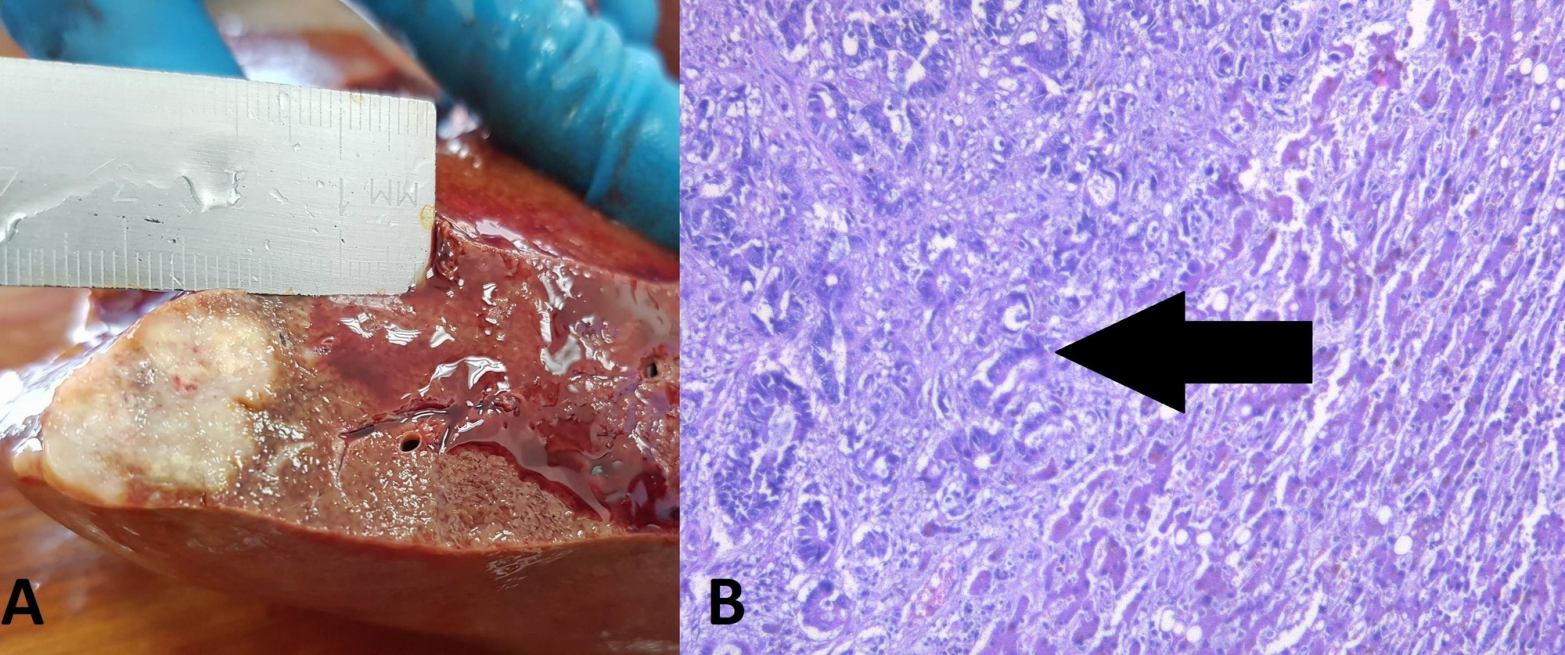
Liver lesion. (A) Autopsy finding; (B) histology: hematoxylin and eosin stain, original magnification 100x, atypical glands with invasive growth into the liver parenchyma (arrow).

Abdominal section revealed a solid sub-capsular tumor formation in the liver measuring 2.5 cm in diameter (Figure 3A).

**Figure 4:**
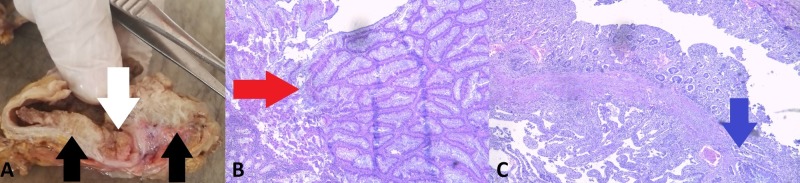
Polypoid findings in the cecum. (A) Autopsy finding: noninfiltrative lesion (white arrow), infiltrative lesions (black arrows); (B) histology of the benign lesion: hematoxylin and eosin stain, original magnification 100x, growth of intestinal glands (red arrow); (C) histology of one of the malignant lesions: hematoxylin and eosin stain, original magnification 100x, growth of atypical intestinal glands infiltrating beyond the muscular layer (blue arrow).

A section of the large intestine revealed multiple polyps, more than 20 in the cecum alone, two of which were with infiltrative growth into the intestinal wall (Figure 4A). The total number of polyps, however, was less than 100.

**Figure 5:**
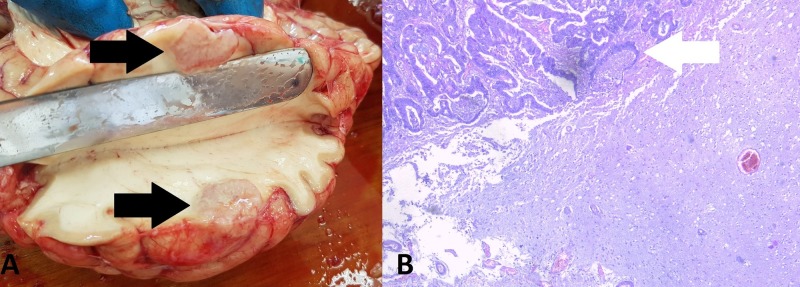
Cerebral lesion. (A) Autopsy finding: section of a well-demarcated formation (arrows); (B) histology: hematoxylin and eosin stain, original magnification 100x, atypical glands with invasive growth into the brain (white arrow).

A section of the cranium revealed a well-demarcated formation, which was 1.8 cm in diameter in the precentral gyrus of the right hemisphere, with well-visible margins from the surrounding brain parenchyma (Figure 5A).

All lesions were composed of atypical epithelial cells, constructing atypical glandular structures, with pronounced mucus production (Figure 1-5B). The difference in appearance in one of the pulmonary masses was due to the excessive mucus production (Figure 2B).

Based on the histological profile of all the lesions (multifocal metastatic colorectal carcinoma pT4NxM1b) the diagnosis of attenuated familial adenomatous polyposis (AFAP) was accepted.

## Discussion

Attenuated familial adenomatous polyposis is a condition in the clinical spectrum of the intestinal polyposis syndromes [4]. This condition is a consolidation of multiple conditions, with different mutations, all presenting with multiple intestinal polyps, less than 100, however, which undergoes malignant transformation after the age of 55.

The current case illustrates the multiformity of the condition. As the patient history is suggestive, but not exclusive for lung cancer and even with a biopsy of the pulmonary lesions, without immunohistochemistry (IHC), the condition would have been still hard to diagnose. Even with the other metastatic locations established and a proper clinical picture, colorectal and lung cancer often share the same metastatic locations and may mimic one another [5].

The current case highlights the importance of a thorough work up to look for a primary tumor source and investigation with tissue diagnosis, as well as the need for investigative testing with colonoscopy to evaluate other common malignancies that can first present with a solitary or multiple lung lesions.

## Conclusions

Attenuated familial adenomatous polyposis is an extremely multiform condition, which can present in a variety of ways, as demonstrated with the current case. IHC is useful in cases of biopsy of the lesion with a phenotype uncommon for the location, as in the current case.
